# Personalized exposure and experience sampling method feedback versus exposure as usual for obsessive–compulsive disorder: a study protocol for a randomized controlled trial

**DOI:** 10.1186/s13063-023-07780-5

**Published:** 2024-01-12

**Authors:** Elena Hoogerwerf, Anja Greeven, Rutger Goekoop, Philip Spinhoven

**Affiliations:** 1 Parnassia Groep Academie, Dadelplein 1, 2552DS The Hague, The Netherlands; 2 Institute of Psychology, Section of Clinical Psychology, Leiden University, Wassenaarseweg 52, 2333 AK Leiden, The Netherlands; 3 Department of Psychiatry, Leiden University Medical Center, Albinusdreef 2, 2333 ZA Leiden, Leiden, the Netherlands

**Keywords:** Obsessive–compulsive disorder, Exposure with response prevention, Experience sampling, Ecological momentary assessment, Ecological momentary interventions, Video-calling CBT, Cognitive behavorial therapy, Personalized medicine, Mediation analysis, Randomized controlled trial

## Abstract

**Background:**

Patients with obsessive–compulsive disorder (OCD) suffer from repetitive fearful intrusions which they try to neutralize by performing compulsions. OCD is considered to be the most resistant anxiety disorder with a remission rate of only 53% after a year of an evidence-based treatment. Therefore, it remains an obligation to develop and investigate more effective treatment interventions. This study aims to compare personalized exposure with response prevention (ERP) using experience sampling methodology-based feedback to ERP as usual in patients with OCD. Personalized exposure will be provided screen-to-screen in an ecologically valid (real time and real place) context by means of a smartphone application. This app will also be used to collect both objective and subjective data by means of experience sampling methodology (ESM). This ESM data will be used to identify triggers and protective factors for symptom severity, provide personalized feedback and optimize the effect of ERP. The primary goal of this RCT is to compare the effectiveness of personalized ERP to ERP as usual in the traditional context of a therapist’s room in patients with OCD in OCD symptom severity, as well as differences in quality of life, depressive symptoms and anxiety states. Since both self-efficacy and experiential avoidance are known to influence symptom severity in OCS, a secondary goal is to examine if a possible treatment effect is mediated by self-efficacy or experiential avoidance.

**Methods:**

This study involves a randomized controlled trial with 20 weekly sessions by 2 groups (ERP as usual versus personalized ERP), repeated measurements at baseline (T0), 5 weeks of treatment (T1), 10 weeks of treatment (T2), 15 weeks of treatment (T3), posttest at 20 weeks (T4), 6 weeks follow-up (T5), 3 months follow-up (T6), 6 months follow-up (T7) and a year follow-up (T8). A hundred and sixty patients with an OCD diagnosis according to DSM-5 criteria will participate. Half of the group will receive exposure with response prevention as usual, the other half will receive personalized exposure with response prevention with a smartphone application and personalized feedback sessions based on experience sampling data. Multilevel mixed modelling analysis will be used to investigate differences in treatment effect, as well as differences in quality of life, depressive symptoms and anxiety states. We will use the macro of Preacher and Hayes and apply bootstrapping methods to assess the possible mediating effect of changes in self-efficacy and experiential avoidance on subsequent treatment effects.

**Discussion:**

This randomized controlled trial is the first to assess the influence of delivering ERP through video-calling and the use of an ESM intervention on the symptom severity of OCD. Since the global pandemic COVID-19, the use of video-calling to deliver psychological treatments has become more common, increasing the relevance of this study.

**Trial registration:**

ICTRP Trial NL8254. Registered on 2019–12-24.

**Supplementary Information:**

The online version contains supplementary material available at 10.1186/s13063-023-07780-5.

## Administrative information

**Table:** 

Title {1}	Personalized exposure and ESM feedback versus exposure as usual for OCD: a study protocol for a randomized controlled trial
Trial registration {2a and 2b}	International Clinical Trial Registry Platform NL8254 https://trialsearch.who.int/Trial2.aspx?TrialID=NL8254
Protocol version {3}	First version 30–10-2019
Funding {4}	No funding
Author details {5a}	^1^ Parnassia Groep Academie, Dadelplein 1, 2552DS Den Haag ^2^ Institute of Psychology, Section of Clinical Psychology, Leiden University, Wassenaarseweg 52, 2333 AK Leiden, The Netherlands ^3^ Department of Psychiatry, Leiden University Medical Center, Albinusdreef 2, 2333 ZA Leiden, Leiden, the Netherlands
Name and contact information for the trial sponsor {5b}	Dr. J. Verbeeck Psyq, Directeur Zorg
Role of sponsor {5c}	The sponsor has no role in study design, analysis, interpretation, or publication of the study protocol and trial results

## Introduction

### Background and rationale {6a}

Patients with obsessive–compulsive disorder (OCD) suffer from repetitive and persistent fearful obsessions or intrusions—at least 1-h a day—which they try to neutralize, ignore, or suppress by performing compulsions. These compulsions can present themselves in the form of repetitive mental acts (covert behaviors) or actions (overt behaviors) [1]. The life-time prevalence of OCD is 2.3%, and its course—when untreated—is mostly chronic with symptoms waxing and waning [2, 3]. The World Health Organization ranks OCD as one of the 10 most burdening disorders in terms of its impact on quality of life [4].

National and international guidelines prescribe exposure and response prevention (ERP) as the first step in a stepped-care approach of OCD, sometimes augmented with cognitive therapy or pharmacological interventions [5]. Treatment is mostly provided in an ambulatory mental health care setting and usually involves weekly sessions. But patients with OCD experience symptoms in a large variety of settings and circumstances. The laboratory-like context of a standard mental health care facility is an impoverished diagnostic and treatment environment, which does not fully do justice to the heterogeneity of OCD symptoms. This opinion is supported by a growing number of studies, which show that high relapse rates and disappointing response rates of exposure seem to be related to defects in extinction learning—among others—related to lack of variation in the way exposure is offered [6, 7].

In clinical practice, OCD is considered as the most resistant anxiety disorder; a finding that is empirically supported by several meta-analyses which show that remission rate of OCD 1 year after conclusion of an evidence-based treatment with a SSRI or a cognitive behavioral approach is about 53% and that treatment effect diminished rapidly after the acute treatment phase [8–10]. These percentages indicate that a substantial part of OCD patients does not adequately respond to the current treatment options. Therefore, it remains a challenge and an obligation to develop and rethink treatments possibilities for patients suffering from OCD to decrease personal suffering and societal costs.

One way to improve care is to tailor treatment to the individual symptom profile of each patient, and to increase the ecological validity of exposure interventions. Making therapeutic efforts more “ecologically valid” may improve the effectiveness of this intervention (e.g., having patients practice with their own locks and windows, or touching their own toilet seat instead of those of the outpatient clinic). The introduction of smartphones and other technologies have opened a world of possibilities to offer exposure treatment outside the therapist’s office. Different studies found that internet-based delivery of exposure with response prevention leads to a clinical significant decrease in symptoms [11–13]. However, there is a need for randomized studies with a face-to-face control group. These findings are confirmed in a meta-analysis which found that delivering OCS treatment via video-conferencing holds promise to be at least equally effective as face-to-face treatment but comparison studies with face-to-face control groups are necessary [14].

We therefore propose a more flexible approach, using the benefits of modern technology, in which online (screen to screen) communication is used to provide treatment in the real time and real world of the patient. Additionally, we will further personalize these interventions by performing experience sampling methodology (ESM: keeping a digital diary, performing repeated self-measurements) to identify specific temporal relationships between the symptoms of OCD and emotional states, which can be selectively targeted in therapy. As a result, exposure exercises will be more precisely tailored to the unique context of the patients’ complaints, optimizing the effect of exposure. Therefore, this study aims to investigate the effect of online (screen-to-screen) and ESM-enhanced exposure with response prevention (ERP) to ERP as usual in patients with OCD.

Furthermore, we will be assessing possible predictors and mediators of treatment effect. Experiential avoidance, the avoidance of unpleasant inner experiences such as fear, discomfort and bodily sensations is found to play a crucial role in the development and maintenance of obsessive–compulsive symptoms [15–19]. Also, meta-analysis showed that this effect differed between the different subtypes of OCD, patients with the focus on obsessions and responsibility for harm seem to benefit more from a reduction in experiential avoidance [17]. Therefore, we will examine the possible effect of experiential avoidance as a predictor for treatment outcome and investigate if this differs between subgroups of patients. Also, self-efficacy, someone’s belief in his or her capacity to perform actions necessary to attain certain goals, has found to be positively correlated with symptom severity in OCD [20, 21]. Moreover, self-efficacy was found to have a mediating role in the treatment of OCS [22]. Since our experimental condition appeals to patients’ own contribution in their treatment approach, we aim to investigate if there is a difference in the increase of self-efficacy between conditions and if self-efficacy works as a mediating factor on treatment effect.

### Objectives {7}

The objectives of this trial are:

- To investigate the effectiveness of personalized exposure and response prevention (ERP) in patients with OCD in improving OCD symptom severity.
- To examine if a possible treatment effect is mediated by experiential avoidance.
- To assess if patients’ feelings of self-efficacy regarding the therapeutic process functions as a mediator on the treatment effect

### Trial design {8}

The design of the study will be 20 sessions (on a weekly basis) by a 2-group (ERP as usual versus personalized ERP) randomized controlled clinical trial with repeated measurements at baseline (T0), at 5 weeks of treatment (T1), at 10 weeks of treatment (T2), at 15 weeks of treatment (T3), posttest at 20 weeks of treatment (T4), 6-week follow-up (T3), 3-month follow-up (T4), 6-month follow-up (T5), and a year follow-up (T6). Assessment will consist of a semi-structured clinical OCD interview and a broad spectrum of questionnaires consisting of personality (trait) and symptom (state) lists. In the experimental condition, ESM data will be gathered throughout the study.

After conclusion of the treatment period, the patients will have a 6-week therapy break to independently apply what has been learned in therapy. After these 6 weeks, patients will be invited for a booster session in which the learned techniques will be repeated and in which it will be evaluated if the patients’ treatment can be concluded or if more treatment is needed (i.e., in case of non-responding or comorbidity). If necessary, patients will be referred for additional help within or outside the department. After this session, patients will enter a naturalistic follow-up period in which they are allowed to seek help the way they would normally do when confronted with an increase of anxious symptoms (e.g., visiting one’s general practitioner or seeking sources of symptom relief, e.g., using pharmacotherapy).

## Methods: participants, interventions and outcomes

### Study setting {9}

The study will be performed at multiple sites of anxiety disorders departments of PsyQ. PsyQ is part of the Parnassia group, a large urban ambulatory mental health organization in the Netherlands. Other departments in the regions of The Hague and Rotterdam will be made aware of the study and will be asked to refer eligible patients.

### Eligibility criteria {10}

To be eligible to participate in this study, a participant must meet all the following criteria: (1) an OCD diagnosis according to DSM-5, (2) not having received any treatment for OCD in the past 3 months, (3) stable medication for at least three months, and (4) willing to refrain from following other treatment for OCD and keep medication stable during the experimental part of the study. When entering the naturalistic follow-up phase, these restrictions will be released.

Our exclusion criteria will only relate to our obligation to offer appropriate care and to guarantee patient safety. Only patients who suffer from severe comorbidity which necessitate other treatment (psychosis, addiction/intoxication) will be excluded from participation in this study. Since the treatment and questionnaires will be in Dutch or English insufficient fluency in the Dutch or English language is also a criterion for exclusion.

All therapists providing treatment are fully licensed to give cognitive behavioral therapy and must complete a training in exposure skills specifically designed for this study. Furthermore, therapist providing the experimental therapy must complete a training in working with the smartphone application NiceDay. All therapists will be supervised 2-weekly by an experienced and licensed therapist.

### Who will take informed consent? {26a}

After their intake interview, all patients with OCD will be asked to participate in the study. Patients will receive verbal information regarding the study and an information letter containing all relevant information. One week after receiving the letter, one of the research assistants will contact the patient by phone to answer possible questions. If the patient decides to participate in the study, the research assistant will schedule a meeting and will make sure informed consent is given with a signed informed consent form.

### Additional consent provisions for collection and use of participant data and biological specimens {26b}

Not applicable, no samples collected.

## Interventions

### Explanation for the choice of comparators {6b}

We chose to compare personalized exposure with exposure as usual because we aim to improve standard of care. Therefore, we want to directly compare personalized exposure with the current treatment of choice, exposure as usual.

### Intervention description {11a}

#### Exposure with response prevention (ERP)

Patients in both conditions will receive exposure with response prevention (ERP), the first step in both national and international treatment guidelines for OCD [5]. ERP can be defined as planned and repeated systematic confrontation with internal and external fear-provoking cues (exposure) combined with refraining from rituals reducing fear (response prevention).There is strong support for the effectiveness of ERP, this was confirmed in a fairly recent meta-analysis [23]. Although traditionally habituation was seen as the primary mechanism in ERP, this focus shifted to inhibitory learning in the past decade [6, 24]. According to this rationale, both unlearning old catastrophic interpretations and learning a wide range of new associations form the key to therapeutic change. Exposure is hypothesized to be effective when the new association becomes stronger than the old association. Within this explanatory model, the context in which the exposure takes place is extremely important. Several studies have shown that learning is context-dependent and that exposure in multiple contexts appears to counteract a return of fear [6, 24].

#### Control condition: exposure with response prevention as usual

Patients in the exposure with response prevention as usual group will be treated according to the current national guidelines for OCD [25]. This means that they will receive ERP as described above. Exposure will take 20 sessions and will be offered on an individual basis. This number of sessions is in line with the current national guidelines [25]. ERP will take place once a week, for 60 min, supplemented with homework exercises which consist of ERP exercises. Sessions will consist of discussion of the homework exercises, in session ERP and preparing homework for the following week. The course and effect of therapy will be monitored by means of the questionnaires which are part of the study.

#### Experimental condition: exposure with response prevention with NiceDay

Patients in the experimental condition will receive ERP as described above but offered through the smartphone app NiceDay. Also, they will receive personalized feedback, based on ESM data gathered with NiceDay, wherein their OCD symptoms will be placed into the context of emotional, cognitive, and motivational states and daily life events. NiceDay is a smartphone application which is developed in close collaboration with mental health care professionals and patients. NiceDay will be used both as a tool which enables video-calling with patients and a data collection tool. NiceDay collects raw data but does not interpret this data or uses it to intervene.

During treatment, patients will have 45-min weekly sessions with their therapists. Sessions will consist of discussion of the homework exercises, in session ERP and preparing homework for the following week. However, in the experimental condition, these sessions will be offered digitally, through video-calling. Exposure exercises will be offered in real time and real world (i.e., the real life, natural context of the patient). Digital sessions offer the possibility that patients can practice exposure (e.g., closing a window or touching a toilet seat) in their own environment while being in direct contact with their therapist, instead of conducting their exercises in a less personal and therefore less anxiety-provoking situation, at the treatment center. This way, interventions can be offered in a much more flexible, relevant, and personalized way. Exposure exercises will be agreed upon in advance but will also be need-driven (“on demand”). When patients encounter a situation in which OCD symptoms are triggered, they can use the app to self-guide themselves through an exposure exercise right away, using the build-in exposure form in the application. Practicing exposure exercises in real time and place is expected to increase the ecological validity of the exercises. This method fits perfectly into the inhibitory learning principles of exposure theory as mentioned previously. Also, the smartphone application collects ESM data regarding emotional states. Based on these data, participants will receive personalized feedback weekly by their therapist. Feedback will be provided both verbally and graphically and will reveal interaction between symptoms (i.e., compulsions and intrusions) and emotional states. The interactions will be determined based on the clinical insight of the therapist in agreement with the vision of the patient (shared-decision making). The primary aim of the feedback is to create awareness regarding interactions between symptoms and the personal context of the patient. If indicated, exposure exercises will be tailored to the specific needs of the participant. For example, if the ESM data indicates that a participant experiences an increase in compulsions in an angry state, exposure exercises will take place in that context. Personalized feedback based on ESM data is new to the field of psychology but several studies have tested the intervention in patients with depression with preliminary positive effects on feelings of empowerment and a decrease in symptoms of depression [26–29].

### Criteria for discontinuing or modifying allocated interventions {11b}

Participants can leave the study at any time for any reason if they wish to do so without any consequences. The investigator can decide to withdraw a participant from the study if participating will jeopardize the participant’s health or safety. If the interventions are a risk for participant health or safety, modifications will be submitted for approval to the accredited METC; after a positive decision from the accredited METC, the changes will be implemented. Furthermore, the investigator will suspend the study if there is sufficient ground that continuation of the study will jeopardize participant health or safety. The investigator will notify the accredited METC without undue delay of a temporary halt including the reason for such an action. The study will be suspended pending a further positive decision by the accredited METC. The investigator will make sure that all subjects are kept informed.

### Strategies to improve adherence to interventions {11c}

All the therapists providing treatment are trained in motivating patients to adhere to treatment protocols. Also, the research assistants will have frequent contact with participants by telephone to help with any questions and to motivate participants to keep following the treatment protocol.

### Relevant concomitant care permitted or prohibited during the trial {11d}

During the active treatment phase of 20 weeks, participants are asked to refrain from any other form of care. After concluding the active treatment phase, a treatment-free period of 6 weeks is followed by a booster session. After the booster session, patients will enter a naturalistic follow-up period in which they are allowed to seek help the way they would normally do when confronted with an increase of anxious symptoms (e.g., visiting one’s general practitioner or seeking sources of symptom relief, e.g., using pharmacotherapy).

### Provisions for post-trial care {30}

After conclusion of the treatment period, the patients will have a 6-week therapy break to apply independently the skills learned in therapy. After these 6 weeks, patients will be invited for a booster session in which the learned techniques will be repeated and in which it will be evaluated if the patients’ treatment can be concluded or if more treatment is needed (i.e., in case of non-responding or comorbidity). If necessary, patients will be referred for additional help within or outside the department.

### Outcomes {12}

#### Primary outcome measure

##### Yale-Brown Obsessive–Compulsive Scale (Y-BOCS)

Primary outcome is a decrease in symptom severity, measured with the Y-BOCS, between the measurements taken at baseline and at posttest.

The Y-BOCS is a semi-structured clinical interview which assesses the severity of obsessions and compulsions separately and is the gold standard to measure OCD severity in clinical practice and scientific research. The Y-BOCS consists of 10 items which are rated on a 4-point Likert scale. The internal consistency and validity of the Y-BOCS have proven to be good. Moreover, the Y-BOCS is sensitive to change. The Y – BOCS has been validated in a Dutch clinical population [30]. The Y-BOCS will be administered at each assessment moment by telephone by trained outcome assessors.

#### Secondary outcome measures

##### The World Health Organization Quality of Life – Bref (WHOQOL-Bref)

The WHOQOL-Bref (26 items) was developed as an international cross-culturally comparable self-report quality of life assessment instrument [31]. It assesses the individual’s perceptions of quality of life in the context of their culture and value systems, personal goals, standards, and concerns across 4 domains: physical health, psychological health, social relationships, and environment. The internal consistency of the four domains of the WHOQOL-Bref ranged from 0.66 to 0.80. Domain scores of the WHOQOL-Bref correlated around 0.92 with the WHOQOL-100 domain scores. Relatively low correlations were found between demographic characteristics (age and sex) and WHOQOL-Bref domain scores. It is concluded that the content validity, construct validity, and the reliability of the WHOQOL-Bref in a population of adult Dutch psychiatric outpatients are good [31]. The WHOQOL will be administered at baseline, midtest, posttest, and all follow-up assessment moments.

##### The 16-item self-report Quick Inventory of Depressive Symptomatology (QIDS)

The QIDS is derived from the 30-item Inventory of Depressive Symptomatology (IDS) and is available in both self-report (QIDS-SR16) and clinician-rated (QIDS-C-16) formats [32]. The internal consistency was high for the QIDS-SR16 (Cronbach’s alpha = 0.86). Furthermore, the QIDS-SR16 is sensitive to symptom change and has highly acceptable psychometric properties [32]. The QIDS-SR16 has been validated in a Dutch clinical population [33]. This scale is included because there is a high comorbidity rate between depression and OCD. The QIDS will be administered at baseline, midtest, posttest, and all follow-up assessment moments.

##### The State-Trait Anxiety Inventory (STAI)

The STAI consists of 2 subscales (state and trait anxiety), each comprising 20 items [34]. Scores range from 20 to 80, with higher scores suggesting greater levels of anxiety. A Dutch validation study of the STAI showed its reliability and sensitivity in the measurement of anxiety (46). The STAI has been validated in a Dutch clinical population [35]. Administration takes about 10 min. This scale is included because there is a high comorbidity rate between anxiety states and OCD. This scale allows for better differential diagnostics and more detailed subgroup definition on our population. The STAI will be administered at baseline, midtest, posttest, and all follow-up assessment moments.

##### Experience sampling data

ESM data will be gathered via NiceDay in the experimental condition. At three random moments per day, participants will be asked to register their emotional states. Participants will be asked to register their compulsions in the app throughout the day. Furthermore, participants can use the dairy option to plan activities, such as exposure exercises. The data collected with NiceDay will be used as an indirect measure to assess patients’ motivation, self-efficacy, and adherence to the treatment, by, for example, the number of registrations, how often a patient takes initiative to plan activities. Participants in the experimental condition will be invited to fill out experience sampling data during the active treatment phase of 20 weeks.

#### Mediation variables

We will be using the following measures to assess for a possible mediating role on treatment effect.

##### The Self-Efficacy Questionnaire for OCD (SEQ-OCD)

In order to measure patient’s self-efficacy regarding their OCD symptoms, we adapted the self-efficacy questionnaire for phobic situations (SEQ-PS). The SEQ-PS is a 13-item psychometric scale that is designed to measure the perceived ability to cope with phobic symptoms when approaching feared stimuli [36]. Responses are recorded on a 5-point scale. The SEQ-SP was found to have Cronbach’s alpha of 0.94. We adapted the SEQ-PS to measure symptoms of OCD when being confronted with the object of intrusions and not being able to perform compulsions. The SEQ-OCD will be included in all measurement moments.

##### The Acceptance and Action Questionnaire II (AAQ-II)

The AAQ-II [37] is a 7-item measure of psychological inflexibility/experiential avoidance. Answers are given on a 7-point scale ranging from 1 = never true to 7 = always true. The Dutch translation of the AAQ-II has been found to have good psychometric properties [38]. Experiential avoidance has been found to play some role in the development of obsessive–compulsive symptoms but it remains yet unclear to what extent [15, 18, 19]. We included the AAQ-II to assess the possible role on symptom severity and treatment effect. The AAQ-II will be included in all measurement moments.

#### Control variables

We will be using the following measures to control for a possible influence on treatment effect.

##### Technology Acceptance Questionnaire (TAQ-NL)

We will use a technology acceptance questionnaire (TAQ-NL) to assess whether acceptance of the NiceDay application influences treatment outcome in the intervention group. The TAQ is adapted from the Technology Acceptance Model and extended with constructs from the Unified theory of Acceptance and Use of Technology [39]. Constructs of the questionnaire include intention of use, perceived ease of use, perceived usefulness, and several organization context factors (i.e., facilitating conditions, subjective norm). The TAQ-NL was pilot tested and is currently being validated in a Dutch sample [40]. The items are presented in statements which will be rated on a 5-point Likert scale ranging from 1 = “strongly disagree” to 5 = “strongly agree”. The TAQ-NL will be administered to participants at posttest.

##### The eHealth Literacy Questionnaire (eHLQ)

To determine whether eHealth Literacy influences treatment effect in the intervention group, we will use the eHealth Literacy Questionnaire. The eHealth Literacy Questionnaire (eHLQ) aims to measure eHealth literacy based on the 7-dimensional eHealth Literacy Framework [41]. The eHLQ consist of 35 items that are scores on a 7-scale answers scale. It consists of 7 scales, namely; using technology to process health information, understanding of health concepts and language, ability to actively engage with digital services, feel safe and in control, motivated to engage with digital services, access to digital services that work and digital services that suit individual needs. The eHLQ was found to have good psychometric abilities [41].

### Participant timeline {13}

**Fig. 1:**
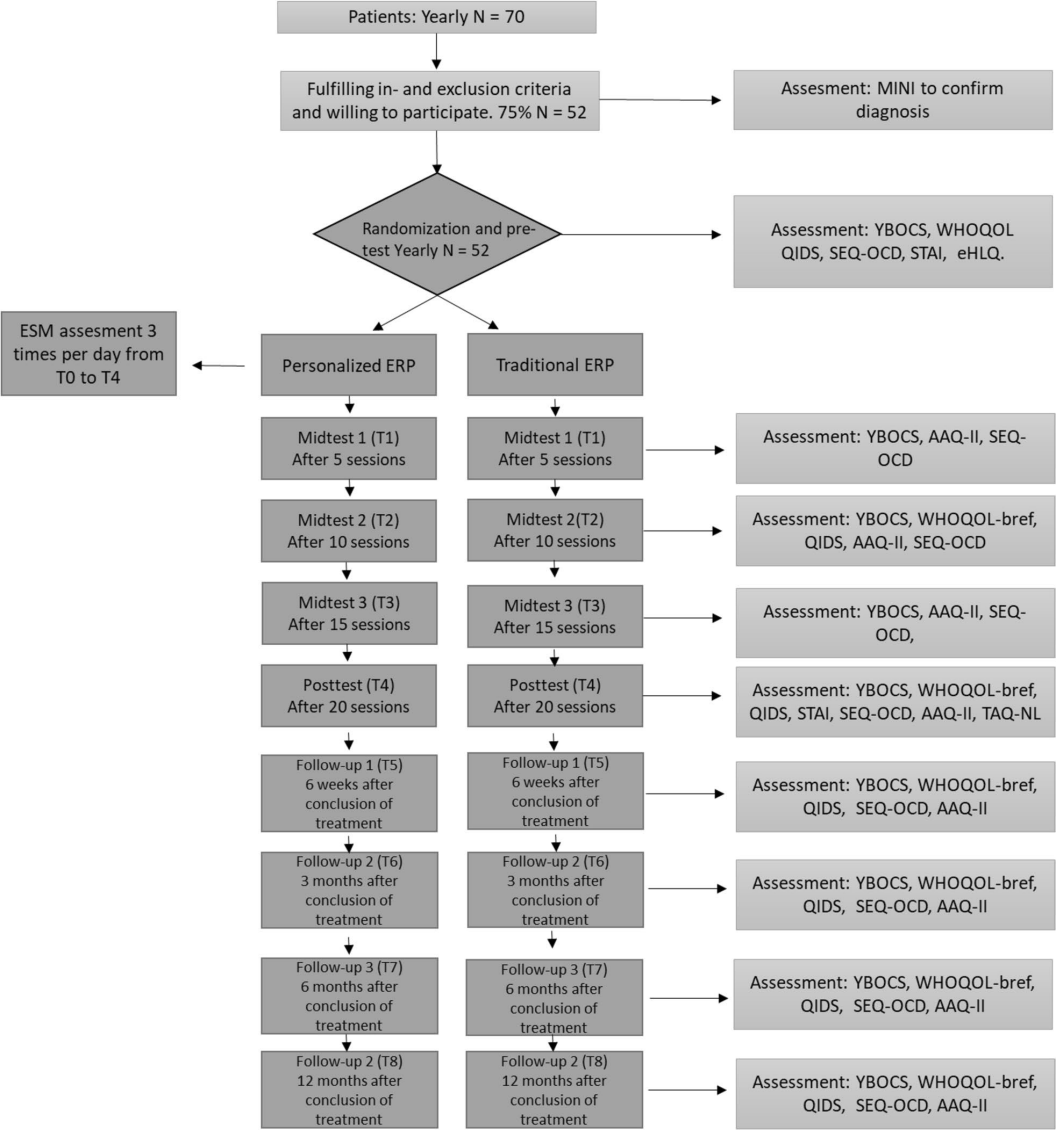
Flowchart of participant timeline. In the context of parallel research goals outside the scope of this paper multiple other questionnaires not mentioned in the flowchart will be administered

**Fig. 2:**
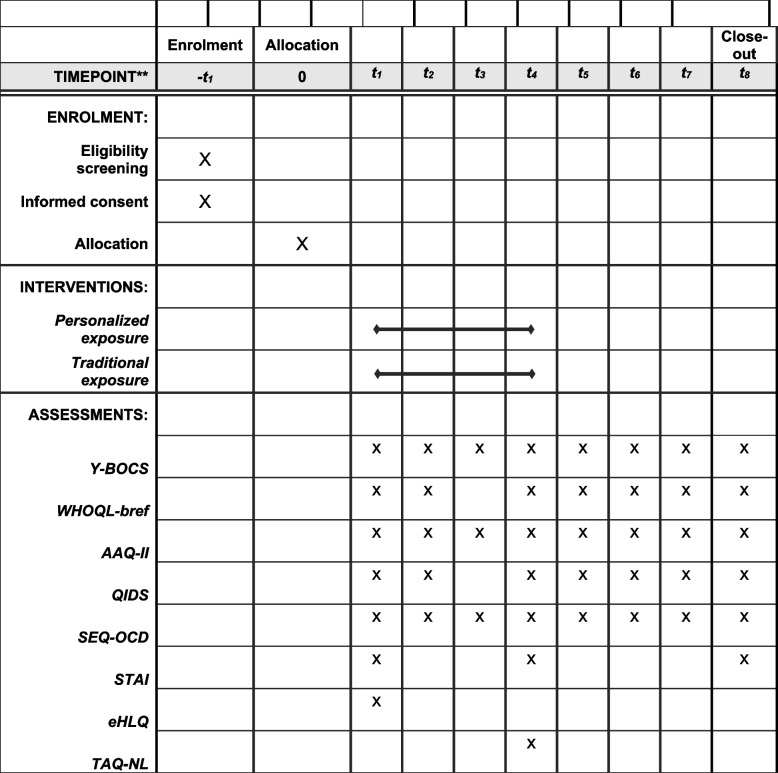
SPIRIT schedule of enrollment, interventions and assessments

Figure 1 shows the participant timeline, and Fig. 2 shows the SPIRIT schedule of enrollment, interventions and assessments.

### Sample size {14}

A statistical power analysis to estimate sample size for longitudinal, correlated data was performed [42, 43]. This analysis revealed that with an alpha of 0.05, an estimated correlation of 0.5 between measurements and a power of 0.85 the sample size needed to detect a small to medium effect (*f* = 0.35) is 160. We expect a small to medium effect since a large effect is unlikely due to the small differences between the conditions. Also, we are not interested in finding a small effect; therefore, we choose to calculate a sample size large enough to detect a clinically relevant effect with a medium effect size. Therefore, we propose a total sample size of 160 patients.

### Recruitment {15}

Participants will be recruited within the different departments of PsyQ, other mental health institutions within the region, and directly through general practitioners. General practitioners, mental health institutions, and possible participants will be informed about the study through social media, patient associations, and word to mouth communication. Recruitment started in the first half of 2020 and will continue until the sample size is reached, which will likely be around July 2024.

## Assignment of interventions: allocation

### Sequence generation {16a}

Patients will be assigned to either (1) ERP as usual or (2) personalized ERP. Scientific literature shows that the prevalence of OCD is not determined by gender (a ratio of 1:1) [44]. However, there are differences in symptomatology between the genders, with women presenting more contamination obsessions and men reporting more obsessions of a sexual nature [45, 46]. Furthermore, males with OCD have an earlier age of onset and being of male gender is a prognostic factor for a poor treatment outcome [10, 45, 47]. Since gender is a prognostic factor of treatment response, randomization will be stratified according to gender. There is no gender difference in the prevalence of OCD so the randomization ratio will be 1:1. The allocation sequence is generated by the software package Research Manager. Research Manager is an online research platform which provides multiple tools to manage clinical trials (e.g., encrypted data storage, randomization tools) [48].

### Concealment mechanism {16b}

The allocation sequence is generated by the software package Research Manager. After generation of the sequence, it is not possible to open the randomization table. Only the research assistants have authorisation to randomize participants. There is a strict protocol which assures that the randomization outcome stays concealed. Only one research assistant is aware of the outcome while the other performs the measurement blinded.

### Implementation {16c}

The allocation sequence was generated by the principal investigator before start of the study. Two research assistants will assign participants to interventions.

## Assignment of interventions: blinding

### Who will be blinded {17a}

Since we are investigating two active treatment conditions, both participants and therapist will not be blinded to assignment. All outcome assessors and data analysts will be blinded for intervention assignment.

### Procedure for unblinding if needed {17b}

Unblinding is only permissible in the very rare case when it is necessary for the participants’ health (e.g., when the researchers need to be involved in discussing other treatment steps for the participant).

## Data collection and management

### Plans for assessment and collection of outcomes {18a}

In our study, we will administer a combination of structured clinical interviews and self-report questionnaires. Clinical interviews will be administered by trained outcome assessors. Outcomes of the questionnaires will be collected through the software package Research Manager and stored in encrypted form on a secured server. Measurement moments will be at baseline, 5 weeks of treatment, 10 weeks of treatment (midtest), 15 weeks of treatment, 20 weeks of treatment (posttest), 6 weeks follow-up, 3 months follow-up, 6 months follow-up, and 12 months follow-up. Primary and mediation outcomes will be measured at all measurement moments. Secondary outcomes will be measured at baseline, midtest, posttest, and in all follow-up moments. Control variables will be measured at baseline. For more information on outcomes, see {12}.

### Plans to promote participant retention and complete follow-up {18b}

Participants will be approached by telephone to motivate them to fill out the questionnaires and to see if any help is needed. When participants decide to drop out of the intervention protocol, the reason for dropping out will be noted and participants will be asked to continue to fill out all the measurements.

### Data management {19}

All involved researchers have a thorough understanding of the AVG (Dutch) and GDPR (European) privacy regulations, are certified regarding Good Clinical Practice (GCP), and will ensure that the research adheres to these standards. Data generated with the NiceDay application will be stored in encrypted form on a secured server owned by Sense Health. Sense Health conforms to the Dutch NEN 7510 and international ISO 27001 standards for information security management. The NiceDay app used in the study also conforms to these standards, and any data processed or stored during this research will be handled in a GDPR compliant fashion. This includes measures for tracking explicit consent, anonymizing or pseudonymising data, and limiting access only to approved research personnel. A full overview of the collected variables and how they will be managed can be provided on request. Outcomes of the questionnaires will be collected through Research Manager and stored in encrypted form on a secured server.

### Confidentiality {27}

A subject identification code list will be used to link the data to subjects. This code will be secured, and the key to the code will be safeguarded by the investigator and senior researchers. The Health Care Inspection (Inspectie Gezondheidszorg en Jeugd) and the internal science committee of the Parnassia.

Groep can request access to the data to ensure the quality of the study. All data will be stored for the legal retention period of 15 years.

### Plans for collection, laboratory evaluation, and storage of biological specimens for genetic or molecular analysis in this trial/future use {33}

Not applicable, no specimens will be collected.

## Statistical methods

### Statistical methods for primary and secondary outcomes {20a}

#### Primary outcomes

Multilevel mixed modelling analysis will be used to investigate differences in treatment effect, measured through symptom reduction on the Y-BOCS. Multilevel analysis is especially suitable to analyze repeated measurement data because it takes into account the dependencies among observations nested within individuals. Another advantage of this methodology is its ability to handle missing data. We will start with a three-level structure with treatment effect (Y-BOCS score) as dependent variable, repeated measures at the first, individuals at the second, and treatment condition at the third level. We will then add age, gender, Ehealth literacy scale scores, and medication use to the model as covariates. We will determine the covariance structure empirically. Effect sizes and clinical significant change according to the Jacobson and Truax criteria will be calculated to estimate respectively the magnitude of the treatment effect and the significance of the results for clinical practice [49]. All analyses will be done both according to the intention-to-treat principle and per protocol in order to gain as much insight as possible into the efficacy of the intervention, as recommended by CONSORT.

#### Secondary outcomes

Multilevel mixed model analysis will be used to assess differences in quality of life, anxiety states, and depressive symptoms between the conditions. We will start with a three-level structure, with respectively quality of life, anxiety states, and depressive symptoms as dependent variable, repeated measures at the first, individuals at the second, and treatment condition at the third level. Age, gender, Ehealth literacy scale scores, and medication use will be added as covariates.

#### Mediation analysis

In order to assess differences between the groups in self-efficacy and experiential avoidance, we will apply multilevel analysis with the scores on the SEQ-OCD and AAQ-II scores at baseline, midtest, and posttest will serve as dependent variables, treatment condition (ERP as usual versus personalized ERP) as a fixed (dichotomous) factor. Furthermore, we will use the macro of Preacher and Hayes and apply bootstrapping methods to assess the possible mediating effect of changes in self-efficacy and experiential avoidance on subsequent treatment effects.

### Interim analyses {21b}

Non applicable, no interim analyses will be done.

### Methods for additional analyses (e.g., subgroup analyses) {20b}

Non applicable, no additional analyses are planned.

### Methods in analysis to handle protocol non-adherence and any statistical methods to handle missing data {20c}

All data will be analyzed according both to the intention-to-treat and per protocol principle in order to gain as much insight as possible into the efficacy of the intervention, as recommended by CONSORT.

### Plans to give access to the full protocol, participant-level data, and statistical code {31c}

We will provide access to participant-level data without any kind of demographic information, to ensure anonymity of the participants. Data and statistical code will be provided by the corresponding author at request.

## Oversight and monitoring

### Composition of the coordinating center and trial steering committee {5d}

This trial is being conducted within PsyQ, center The Hague. PsyQ is part of the Parnassia Groep, a large urban ambulatory mental health organization in the Netherlands. The study will be monitored once a year according to the Standard Operating Procedure monitoring within the Parnassia Groep. PsyQ has appointed a Research Committee (CWO) to oversee all scientific studies in the organization. The CWO consists of multiple researchers, at least one professor, a methodologist, and a manager responsible for the quality of care. The committee gathers every 3 months. All studies conducted within PsyQ are monitored once a year to ensure the quality of medical-ethical concerns, the storage of data, and progression of recruitment. In addition, the Parnassia Groep has appointed a Central Research Committee (CCOI) who is responsible for quality assessment of all studies within the Parnassia Groep. Once a year, this committee will consult the local CWO in regard to quality assessment and progress regarding the ongoing studies.

### Composition of the data monitoring committee, its role and reporting structure {21a}

Not applicable. Since our treatment is not expected to be harmful and all our participants are likely able to express themselves, no data safety monitoring board is established.

### Adverse event reporting and harms {22}

No serious adverse events are to be expected, but when a serious adverse event occurs the same procedures will be followed as in routine treatment. Depending on the seriousness of the adverse events, measures will be taken. In the current project, 1 psychiatrist per department is connected to the study who can be consulted in case of emergencies.

The investigator will report all SAEs to the sponsor without undue delay after obtaining knowledge of the events. The sponsor will report the SAEs through the web portal *ToetsingOnline* to the accredited METC that approved the protocol, within 7 days of first knowledge for SAEs that result in death or are life threatening followed by a period of maximum of 8 days to complete the initial preliminary report. All other SAEs will be reported within a period of maximum 15 days after the sponsor has first knowledge of the serious adverse events.

### Frequency and plans for auditing trial conduct {23}

The sponsor/investigator will submit a summary of the progress of the trial to the accredited METC once a year. Information will be provided on the date of inclusion of the first subject, numbers of subjects included, and numbers of subjects that have completed the trial, serious adverse events/serious adverse reactions, other problems, and amendments.

### Plans for communicating important protocol amendments to relevant parties (e.g., trial participants, ethical committees) {25}

Amendments are changes made to the research after a favorable opinion by the accredited METC has been given. All amendments will be notified to the METC and updated in the trial register.

### Dissemination plans {31a}

The results of this trial will be published in peer-reviewed journals and presented on relevant conventions. Also, a summary of the results will be sent to all participants.

## Discussion

This paper described the study protocol of an RCT comparing traditional exposure to personalized exposure. Personalized exposure will be offered through video-calling in the environment of the participant, with the intention to increase the ecological validity of the ERP. ESM data will be collected and used as an intervention to personalize the exposure further. We will compare personalized exposure with exposure as usual, provided in the treatment room of the therapist.

## Limitations

There are some limitations to consider.

- Since our study is combining two interventions in the experimental condition, both the use of video-calling and an ESM intervention, a possible effect cannot be attributed to either one of the interventions. We believe that the severity and persistence of OCD justifies our choice for this so-called “black-box design”.
- Participants are not blinded for treatment allocation when filling out baseline measures, making them susceptible for attrition bias. However, to date, there has been almost no attrition pre-treatment (*N* = 1), and this attrition could not be attributed to treatment allocation.
- There is no waitlist condition in our design. Although there are many arguments against using a non-active condition, the lack of it may influence the internal validity of this study. If we find a possible effect, it cannot be determined to what extent the factor time has influenced the symptoms of participants. We do however believe that the risk of this is minimal since OCD is such a persistent disease with relatively stable symptom strength over time.
- Although one of our inclusion criteria is that possible medication use needs to be stable for 12 weeks, we did not gather information regarding the dosage being used. Since patients with OCD need higher doses of medication compared to other anxiety disorders, this lack of information may compromise the internal validity of this study [50]. We do however believe that this risk is minimal since we control for medication use (yes/no) in our statistical model.

## Strengths

The high relapse rates in patients with OCD make it a necessary challenge to develop and examine alternative treatment methods for this population. To our knowledge, this is the first study to assess the effect of using video-calling and ESM data as an intervention in participants with OCD. Since the global pandemic of COVID-19, the use of video-calling to deliver psychological treatments has become much more common, making our study even more relevant. The sample size of 160 patients and our lenient inclusion criteria increases the ecological validity of the trial. With this study, we aim to contribute to improving the quality of life of patients with OCD.

## Trial status

This trial is registered in the Netherlands trial register on December 19, 2019. Initial recruitment started in January 2020; however, it had to be halted due to COVID–19 measures in the Netherlands. Due the fact that all participants in the face-to-face control condition received treatment via video-calling, almost all data collected between January 2020 and May 2020 was not usable; therefore, inclusion was restarted with a new sample in June 2020. To date, 102 participants have been included, we anticipate to reach the full sample size around July 2024.

### Supplementary Information

**Additional file 1**: **Appendix 1.** Information sheet and consent form. **Appendix A.** Contact details of data participants. **Appendix B.** Overview of measurements. **Appendix C.** Consent Form.

## Data Availability

The PhD student and senior researchers will have access to the final dataset.

## Abbreviations

AAQ-II: Acceptance and Action Questionnaire—II
AVG: Algemene verordening gegevensbescherming (AVG)
CWO: Commissie Wetenschappelijk Onderzoek
CCOI: Centrale Commissie Onderzoek en Innovatie
DSM-5: Diagnostic and Statistical Manual—5
eHLQ: EHealth Literacy Questionnaire
ERP: Exposure with Response Prevention
ESM: Experience Sampling Method
GDRP: General Data Protection Regulation (GDPR)
METC: Medisch Ethische Toetsings Commissie
MSc: Master of science
OCD: Obsessive-compulsive disorder
QIDS: Quick Inventory of Depressive Symptomatology
RCT: Randomized controlled trial
SEQ-OCD: Self-Efficacy Questionnaire for Obsessive–Compulsive Disorder
SEQ-PS: Self-Efficacy Questionnaire for Phobic Situations
STAI: The State-Trait Anxiety Inventory
TAQ-NL: Technology Acceptance Questionnaire—Nederlands
WHOQOL-Bref: World Health Organization Quality of Life Questionnaire—BREF
Y-BOCS: Yale Brown Obsessive–Compulsive Scale
