# Supplementary material for: Personalized exposure and experience sampling method feedback versus exposure as usual for obsessive–compulsive disorder: a study protocol for a randomized controlled trial
Source: Trials. 2024 Jan 12;25:43. doi: 10.1186/s13063-023-07780-5 (PMC10785525; doi:10.1186/s13063-023-07780-5)
Supplement: Supplementary file 1 — Additional file 1: Appendix 1. Information sheet and consent form. Appendix A. Contact details of data participants. Appendix B. Overview of measurements. Appendix C. Consent Form. [file 13063_2023_7780_MOESM1_ESM.docx]

Appendix 1

Information sheet and consent form

**Participant information for participation in medical-scientific research**

**The 3D research: Doorbreek Dwang Digitaal**

**Personalized exposure versus traditional exposure for patients with OCD**.

Dear Sir / Madam,

We are currently conducting a medical-scientific research into the treatment of obsessive-compulsive disorder. You are receiving this information letter because you have been diagnosed with obsessive-compulsive disorder and we would like to ask you to participate in this study. Participation is voluntary. Your written permission is required to participate. Before you decide whether you want to participate in this study, you will receive an explanation of what the study entails. Read this information carefully and ask the researcher for further explanation if you have any questions. You can also ask the independent physician, mentioned at the end of this letter, for additional information. Or you can talk about it with your partner, friends or family. Further information about participating in such a survey can be found at [www.rijksoverheid.nl/mensenonderzoek](http://www.rijksoverheid.nl/mensenonderzoek) (this information is only available in Dutch).

**1. General information**

This research is conducted by PsyQ – TopGGZ Anxiety Disorders Department –

Haaglanden and Leiden University. For this study we are looking for 160 patients who are willing to participate. The Leiden – The Hague – Delft Medical Ethics Review Committee has approved this study. General information about the assessment of research can be found at [www.rijksoverheid.nl/mensenonderzoek (](http://www.rijksoverheid.nl/mensenonderzoek)this information is only available in Dutch).

**2. Aim of the study**

The aim of this study is to determine whether tailored exposure has a better effect than traditional exposure on the treatment of obsessive-compulsive disorder (OCD). From now on we will use the abbreviation OCD or the words obsessive compulsions when talking about obsessive-compulsive disorder. Exposure is a treatment that has been proven to work for the treatment of compulsive symptoms. Paragraph 3 will provide more information about the content of this treatment.

**3. Background of the study**

There are effective treatments for people with an obsessive compulsive disorder (OCD). Currently, exposure is the treatment of choice for OCD. During an exposure treatment patients are asked to expose themselves to their fears and to refrain themselves from acting on their compulsions. In this way the pattern of obsessions and compulsions is broken. Prior research has shown that many patients with OCD recover during treatment. However, research also shows that there is a large group of patients who show insufficient results to this treatment or who relapse. We believe that one of the reasons hereof is that the treatment is sometimes insufficiently adjusted to the personal situation of patients. OCD symptoms can have a wide variation. They arise in different situations, in different forms and at different times. Because traditional treatment is often offered at fixed times in the treatment room of the therapist, and not in the patient’s own environment – in which the symptoms are often the most severe- it is possible that the traditional treatment insufficiently applies to the patients personal situation. The aim of this study is to explore whether personalized exposure is more effective than traditional treatment. We will adjust the exposure by offering treatment in the patient’s own environment through a smartphone application. With this application digital exposure can be offered where and whenever you need support. For example, you can facetime or chat with your therapist while you expose yourself to daily life situations, or even when the exposure occurs spontaneously. In addition you can keep track of your progress by registering your emotions, behaviours and environmental influences over the course of your treatment. This information will also be used to enhance the treatment process. In the therapy sessions you and your therapist will use this data to see to which extend these different factors influence your OCD symptoms. We will compare this personalized treatment to traditional treatment (exposure as usual). In the traditional treatment condition you will have weekly exposure sessions at the PsyQ building on Lijnbaan 4.

**4. What participating entails**

If you decide to participate, the treatment phase will take 20 weeks. In those 20 weeks you will be treated with exposure. Half of the participants will get personalized exposure, the other half will get traditional exposure. A draw determines which treatment you will receive. After the research treatment an evaluation meeting will take place to see how you are doing and whether you have further needs for treatment. Then, together with your therapist, you can decide on further treatment steps.

**Visits and measurements**

For the study it is necessary that you have a treatment session once a week. A session lasts 45 minutes to 1 hour. If you are in the personalized exposure condition, you will be offered the treatment digitally by means of a smartphone application. With this application you can facetime and chat with your therapist. During your treatment, if you wish, you can have two appointments at PsyQ. Between appointments, you practice at home with homework assignments that you register in the app. You can also keep track of your progress by registering your emotions, behaviour and various environmental factors in the app. In the traditional condition, you come to PsyQ every week for treatment sessions. In between sessions you practice at home by making homework assignments. In the sessions- in the personalized group as well as in the traditional treatment group- the following will happen:

- The homework assignments from the previous week will be discussed
- Together with the therapist exposure exercises will be done
- Homework assignments for the following week will be discussed

Extra in the personalized exposure group:

Your therapist will discuss the information registered with the smartphone application. You and your therapist examine to what extent the various factors in your daily life influence your compulsive symptoms.

As part of the research, we collect information from you at various times. We do this so that we can monitor how you are doing. This is of great importance for the research. **Appendix C** contains an overview of all measurements taken during the study. In total you will be called at home 9 different times. You will then be asked questions about your compulsive symptoms. A phone call takes approximately 20 to 30 minutes. In addition you will also receive 9 digital questionnaires at different times. The questions are about the course of your complaints. Filling in the first questionnaire will take you about 1 hour, the other questionnaires will take you 10-30 minutes. The questionnaires can be completed in parts.

**Computer task**

If you participate in the study, we will ask you to do a one-time task on a laptop. This task will take place when you have an interview with the research assistant. In this computer task, you will play a simple game in which you will learn the best way to catch a coin with a bucket.

Participation in this task does not involve any risks, but will only cost you some of your time. This task takes 25 minutes, plus 15 minutes of instructions. The task itself does not directly benefit you, but your participation does contribute to new scientific knowledge.

The behaviour we infer from the task can teach us something about which patients with OCD may benefit more from treatment with the smartphone application. In the future we hope that with this knowledge we can better predict which treatment is suitable for which patient.

Taking the task is not mandatory, you will give separate permission for this on the permission form. In other words, you can decide to participate in the study without participating in this task.

**5. What is expected of you?**

Because it is important that our study produces results that will allow us to further improve care for patients with OCD in the future, it is important that you keep a number of agreements in order to participate.

The agreements are that you:

- Do the treatment and homework according to the explanation.
- Do not participate in other medical scientific studies.
- Come to treatment appointments.
- Do not follow any other treatments for compulsive symptoms.
- Keep any medication use stable and don’t start new medication.
- Fill in the various measurements that are part of the research (in appendix C you

will find a complete overview of all measurements)

It is important that you contact the researcher:

- before taking any other medicines. Even if they are homeopathic medicines,

natural medicines, vitamins and/or medicines from the drugstore.

- if you no longer wish to participate in the study.
- if your contact details change.

**6. Potential Adverse Effects and Discomforts**

To the best of our knowledge, there are no risks or side effects associated with personalized exposure. The only extra time it takes is filling in the questionnaires for the study. There is no monetary reward for participating.

**7. Potential Pros and Cons**

It is important that you carefully consider the possible advantages and disadvantages before you decide to participate.

Benefits of participating in the study may include:

- Exposure is a good way to treat compulsive symptoms.
- Personalized exposure as well as traditional exposure can lead to fewer compulsive complaints.
- You have a 50% chance of following a treatment in which exposure is offered in

your own living environment.

- You contribute to scientific knowledge about compulsive complaints and their treatment.

Disadvantages of participating in the study may include:

- You spend extra time filling in the questionnaires.

Participation in the study also means:

- That you have agreements that you must keep (but of course this also applies to regular treatments outside the research)

**If you do not wish to participate or if you want to stop participating in the study**

You decide whether you want to participate in the study. Participation is voluntary. If you do not want to participate, you will be treated for your compulsive symptoms in the usual way. The research assistant or your intaker can provide more information about this.

If you do decide to participate, you can always change your mind and stop anyway, even during the study. You will then be treated in the usual way for your compulsive symptoms. You don't have to say why you're stopping. But if you are stopping you must report this to the researcher immediately. The data collected up to that point will be used for the research.

If there is new information about the study that is important to you, the researcher will let you know. You will then be asked if you want to continue participating.

**8. End of the research**

Your participation in the study will end if:

- all treatment appointments as described under point 4 have taken place
- all measurements as described under point 4 have been filled out
- you choose to stop
- PsyQ, the government or the reviewing medical-ethical review committee, decides to stop the study.

The entire study ends when all participants have completed filling in all measurements. After completing the active treatment phase of the study, your primary care provider will discuss options for further care with you, if appropriate. If you have residual complaints or other complaints after the examination, follow-up treatment in our department is possible.

If you are entered into the personalized exposure group, this form of treatment is only available during the duration of the study

After processing all the data, the researcher will inform you about the most important results of the study. This will happen a few years after your participation, once all outcomes have been researched and written down.

**9. Use and storage of your data**

For this research your personal data will be collected, used and stored. This concerns data such as your name, address, date of birth and data about your treatment. The collection, use and storage of your data is necessary to answer the questions posed in this study and to publish the results. We ask for your permission for the use of your data.

**Confidentiality of your data**

To protect your privacy, your data is given a code. Your name and other information that can directly identify you are omitted. Data can only be traced back to you with the key of the code. The key to the code will be safely stored at the PsyQ location on Lijnbaan in The Hague. The data used for reports or publication only contains the code, but not your name or other data that identifies you.

**Access to your data for monitoring**

Some people can access all of your data. Also the data without code. This is necessary to be able to check whether the research has been carried out properly and reliably. Persons who have access to your data are: the controller/monitor who works for the researcher/client of the investigation works, national and international supervisory authorities, for example the Health and Youth Care Inspectorate. They keep your details hidden. We ask you to give permission for this insight into your data.

**Data retention period**

Your data must be saved for 15 years by PsyQ, Anxiety Disorders Department.

**Preservation and use of the data**

After this research, your data may also be important for other scientific research in the field of compulsive complaints and/or the further development of personalized exposure or the smartphone application. For this, your data will be stored for 15 years. You can indicate on the consent form whether or not you agree to this. If you do not agree to this, you can still participate in the current study.

**Future approach for other studies**

It is possible that we contact you to participate in follow-up research on OCD, personalized exposure or the smartphone application. You can indicate on the consent form whether or not you agree to this. If you do not agree to this, you can still participate in the current study.

**Information about unexpected findings**

During this research, something may accidentally be found that is not important for the research, but is important for you. If this is important for your health, you will be informed by your practitioner. You also give permission for this.

**Revoking consent**

You can always withdraw your consent to the use of your personal data. This applies to this study, but also to the storage and use of your data for future research. The research data collected up to the moment you withdraw your consent will be used in the research

**More information about your rights when processing data**

For general information about your rights when processing your personal data, you can consult the website of the Dutch Data Protection Authority.

The Parnassia Group is responsible for processing your personal data. If you have any questions about your rights, please contact the person who is ultimately responsible for the research. For this research, this is:

Dr. A.T. Spijker

PsyQ, Director of healthcare

See appendix A for contact information

If you have any questions or complaints about the processing of your personal data, we recommend that you first contact PsyQ, Anxiety Disorders Department. You can also contact the Data Protection Officer of the PsyQ institution or the Dutch Data Protection Authority of the central government.

**Registration of the study**

Information about this study is also included in an overview of medical scientific studies, namely the Netherlands Trial Register (https://www.trialregister.nl/). It does not contain any data that can be traced back to you. After the research, the website may display a summary of the results of this study.

**10. Insurance for test participants**

If you participate in the study, there are no additional risks. The Parnassia Group therefore asks the METC not to take out additional insurance.

**11. Inform General practitioner**

We always send your general practitioner a letter to let them know that you are participating in the study.

**12. No Compensation for Participating**

The treatment for the research will not cost you anything extra. You may have to incur costs in the form of the compulsory deductible of your health insurance policy. These costs must also be incurred for standard treatment of compulsive complaints. You will not be paid for participating in this study.

**13. Do you have any questions?**

If you have any questions, please contact the research team. For independent advice about participating in this study, you can contact the independent doctor. He knows a lot about the research, but has no interest in it.

If you have any complaints about the study, you can discuss this with the researcher or your practitioner. If you prefer not to or if this is not possible, you can contact the PsyQ complaints committee. All details for this can be found in Appendix A: Contact details.

**14. Signing consent form**

Two weeks after you have had the interview with the research assistant, you will be asked to decide whether to participate in this research. If you give permission, we will ask you to confirm this in writing on the accompanying statement of consent. By your written consent, you indicate that you understood the information and agree to participate in the study.

Both you and the researcher will receive a signed version of this consent form.

Thank you for your attention.

**16. Appendices to this information**

1. Contact details
2. Schedule of investigative actions / description of investigative actions
3. Consent Form

**Appendix A: contact details of data participants**

Senior researcher:

Dr. J.F Greeven

T: 088-3572759

E: a.greeven@psyq.nl

Executive researcher:

E.M. Hoogerwerf (Msc)

T: 088-3572759

E: elena.hoogerwerf@psyq.nl

Independent doctor:

Dr. M. van den Boogaard, Psychiater

T: 088-3572759

E: m.vandenboogaard@psyq.nl

Complaints:

Monsterseweg 93,

2553 RJ Den Haag

T: 088-3570080 of 088-3570027

E: klachtencie.patientenhaaglanden@parnassiagroep.nl

For more information about your rights:

Parnassia Group Data Protection Officer:

Martin van Rijswijk

T: 088 – 357 1 357

E: fg@parnassiagroep.nl

Therapist:

After you have been drawn into the study, you will be assigned a therapist. It will differ per therapist how and when they can be reached. Your therapist will discuss with you how to maintain contact between sessions.

**Appendix B – Overview of measurements**

This chart provides an overview of the various moments in the research at which we take measurements from you.

This table provides an overview of the time spend per measurement


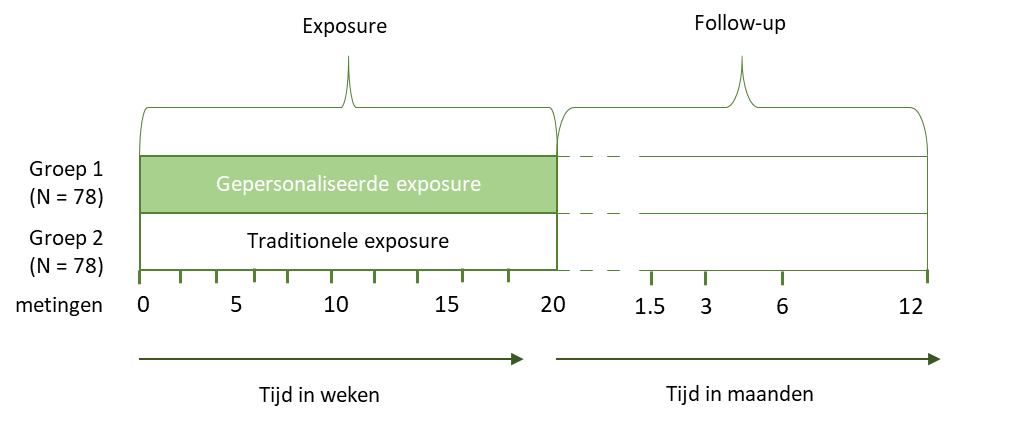


| Moment of Measurement | Time spend in minutes |  |
| --- | --- | --- |
|  | Digital questionnaire | Telephone survey |
| Before start of treatment | 60 minutes | 20-30 minutes |
| 5 weeks of treatment | 10 minutes | 20-30 minutes |
| 10 weeks of treatment | 25 minutes | 20-30 minutes |
| 15 weeks of treatment | 10 minutes | 20-30 minutes |
| 20 weeks of treatment | 30 minutes | 20-30 minutes |
| 6 weeks after the end of treatment | 25 minutes | 20-30 minutes |
| 3 months after the end of treatment | 25 minutes | 20-30 minutes |
| 6 months after the end of treatment | 25 minutes | 20-30 minutes |
| 1 year after the end of treatment | 30 minutes | 20-30 minutes |

**Appendix C: Consent Form**

**The 3D research: Doorbreek Dwang Digitaal**

- I've read the information letter. I also had the possibility to ask questions. My questions have been sufficiently answered. I had plenty of time to decide whether to participate.
- I know that taking part is voluntary. I also know that I can decide at any time not to participate or to stop the study. I don't have to give a reason for that.
- I give permission to inform my general practitioner that I am participating in this study.
- I give permission for the collection and use of my data to answer the research question in this study.
- I know that for the purpose of checking the research, some people may have access to all my data. These people are listed in this information letter. I give permission for access by these individuals.

- I give □ Permission □ No permission

to keep my personal data longer and use it for future research in the field of compulsive complaints, personalised exposure or research about the NiceDay application.

- I give □ Permission

□ No permission

For completing the computer task

- I give □ Permission

□ No permission to approach me again after this research for a follow-up research.

- I want to participate in this study:

Name participant:

Signature: Date: __ / __ / __

--------------------------------------------------------------------------------------------------------------------------------------

I declare that I have fully informed the participant about the said study.

If information becomes known during the study that could influence the participants consent, I will inform him/her in good time.

Name researcher (or it’s representative):

Signature: Date: __ / __ / __

* Strike out what does not apply

*The participant receives a complete information letter, together with a signed version of the consent form.*
